# Myopathy associated with homozygous *PYROXD1* pathogenic variants detected by genome sequencing

**DOI:** 10.1111/neup.12641

**Published:** 2020-02-09

**Authors:** Jeremy D. Woods, Negar Khanlou, Hane Lee, Rebecca Signer, Perry Shieh, Johnathan Chen, Matthew Herzog, Christina Palmer, Julian Martinez‐Agosto, Undiagnosed Diseases Network, Stanley F. Nelson

**Affiliations:** ^1^ Department of Pediatrics University of California Los Angeles Los Angeles California USA; ^2^ Department of Pathology University of California Los Angeles Los Angeles California USA; ^3^ Department of Human Genetics University of California Los Angeles Los Angeles California USA; ^4^ Department of Neurology University of California Los Angeles Los Angeles California USA; ^5^ Department of Radiology University of California Los Angeles Los Angeles California USA; ^6^ Department of Psychiatry and Behavioral Sciences University of California Los Angeles Los Angeles California USA

**Keywords:** genome sequencing, lobulate myopathy, rare disease, trabecular myopathy, undiagnosed diseases network

## Abstract

Biallelic pathogenic variants in the gene *PYROXD1* have recently been described to cause early‐onset autosomal recessive myopathy. Myopathy associated with *PYROXD1* pathogenic variants is rare and reported in only 17 individuals. Known pathogenic variants in *PYROXD1* include missense, insertion and essential splice‐site variants. Here we describe a consanguineous family of individuals affected with late‐onset myopathy and homozygous *PYROXD1* missense variants (NM_024854.5:c.464A>G [p.Asn155Ser]) expanding our understanding of the possible disease phenotypes of *PYROXD1*‐associated myopathy.

## INTRODUCTION

Pathogenic variants in *PYROXD1* are associated with a rare form of progressive and predominantly proximal myopathy inherited in an autosomal recessive pattern (MIM: 617220). *PYROXD1* encodes for a nuclear and cytoplasmic oxidoreductase that has only recently been implicated in human disease with O'Grady *et al*., reporting the first cases of *PYROXD1*‐associated myopathy in 2016.^1^ O'Grady *et al*. established that the p.Asn155Ser allele significantly diminishes reductase activity in *Saccharomyces cerevisiae* cells transformed to express the *PYROXD1* p.Asn155Ser allele.^1^ *PYROXD1*‐associated myopathy was initially characterized by a distinctive histopathology combining features of central and minicore disease as well as centronuclear, myofibrillar and nemaline myopathies.^1^ Myopathy associated with *PYROXD1* pathogenic variants was initially described with an onset in infancy or childhood, although adult‐onset myopathy has also been reported (Table S1).^1^, ^2^, ^3^, ^4^ Cardiomyopathy and restrictive lung disease have also been reported in *PYROXD1*‐associated myopathy, although these phenotypes are variable.^1^, ^2^, ^3^, ^4^

In the previously reported and unrelated families affected by myopathy, both compound heterozygous and homozygous variants in *PYROXD1* including loss‐of‐function variants were reported. Causal variants identified to date include splice‐site mutations (c.285+1G>A and c.414+1G>A), missense variants (p.Asn155Ser, p.Tyr354 and p.Gly372His), frameshift variants (c.1159_1160insCAAA, p.Ala387fs*13) and a deep intronic variant (c.415‐976A>G).^1^, ^2^, ^3^, ^4^ The c.464A>G single nucleotide variant that is predicted to result in a nonsynonymous change p.Asn155Ser is observed in multiple independent families.

*PYROXD1‐*associated myopathy is rare with only 17 cases reported. This paper extends the phenotypic description of *PYROXD1*‐associated myopathy based on three additional affected individuals with homozygous p.Asn155Ser variants with adult‐onset of muscle weakness.

## CLINICAL SUMMARY

**Figure 1:**
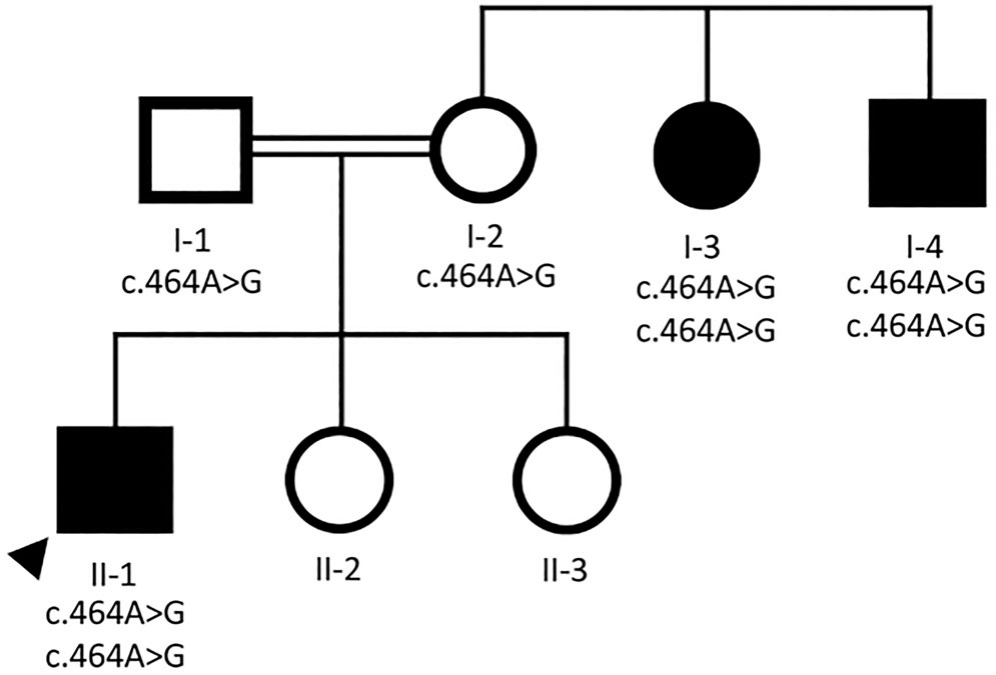
Pedigree of the affected family of Persian‐Jewish heritage. Proband (II‐1) presented to medical attention in his 30s with weakness and had a muscle biopsy at 37 consistent with a myopathic process (see Fig. 3). Patients I‐3 and I‐4 were the less severely affected maternal aunt and uncle of the proband. All affected family members were homozygous for the *PYROXD1* c.464A>G variant.

Three affected patients were identified in a family of Persian‐Jewish descent. The family resided in a remote region of Iran for many generations. Patient II‐1 (proband) was the maternal nephew of patients I‐3 and I‐4 (Fig. 1). Patient II‐1 presented with slowly progressive proximal muscle weakness beginning between age 30–33 years. By the age of 37 he sought medical evaluation because of difficulty climbing stairs and rising from a sitting position for at least 3 years. He also noted weakness when throwing balls. He had no muscle pain or cramping and denied voice changes. He reported that his childhood history was normal with participation in sports without difficulty, but in retrospect was not as strong or as active as others. He was prescribed steroids in his teens for being “thin” although he stopped steroids in his early 20s. Several years after the onset of muscle weakness, he also experienced muscle pain and cramping, especially in his calves after physical activity. His right thigh became increasingly painful even at rest as his disease progressed. Several years after the onset of proximal muscle weakness he developed difficulty swallowing food and would sometimes bite his tongue while chewing. He did not have problems with his voice or problems with his vision by 46 years of age. He remains ambulatory at age 46 years. Patients I‐3 and I‐4 had similar phenotypes as Patient II‐1, but with later onset and a milder course. Patients I‐3 and I‐4 developed muscle weakness in their late 50s which was slowly progressive with difficulty walking, difficulty climbing stairs, difficulty rising from a chair, and muscle cramps. At 82 and 78 years of age, they are without cardiac symptoms or complaints of respiratory failure and remain ambulatory.

Patient II‐1 had initial examination by a neuromuscular specialist at 37 years of age which demonstrated normal tone but decreased muscle bulk. Neck flexor strength was 3/5, deltoids 4+/5, biceps 5/5, triceps 4/5, grip 5/5, interossei 4/5, wrist extensors 5/5, hip flexors 4/5, hip extensors 4+/5, hip abduction 5−/5, hip adduction 5/5, hamstrings 5−/5, quadriceps 5−/5, foot eversion 5/5 and foot inversion 5/5. Scapular winging was present on the right. There was no ataxia, nasal speech, myotonia, or facial weakness. The patient had a normal gait and could even jog a short distance, although he had difficulty rising from a seated position. Sensation was intact. Electromyogram was consistent with a mild non‐irritable myopathy. His proximal muscle weakness slowly progressed, and he developed an intention tremor and waddling gait.

**Figure 2:**
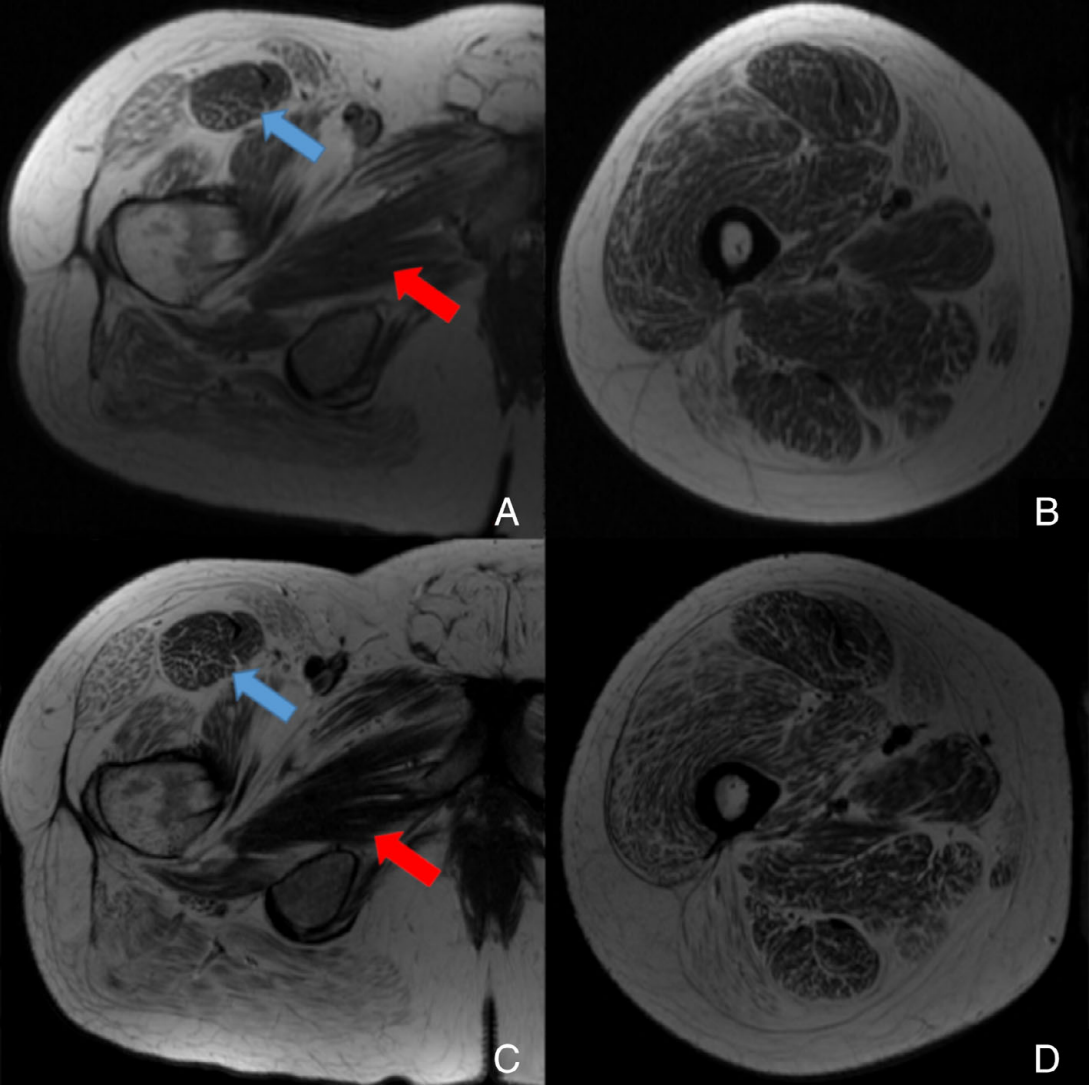
T1‐weighted images on MRI of the thigh muscles. Upper images show Patient II‐1's right proximal (A) and distal (B) thigh at 37 years of age obtained on a Siemans 1.5 T MRI unit. The thigh demonstrates fatty atrophy of all muscular compartments although there is relative sparing of the rectus femoris (blue arrows) and thigh adductor group (red arrows). Lower images show Patient II‐1' right proximal (C) and distal (D) thigh at 46 years old of age obtained on a Siemens 3.0 T MRI unit. The thigh demonstrates fatty atrophy of all muscular compartments. Note that the amount of fatty infiltration since the examination at 37 years of age has increased.

Magnetic resonance imaging (MRI) of the right thigh at 37 years old demonstrated diffuse muscle atrophy that was less prominent in the proximal rectus femoris and the adductor muscles (Fig. 2). His serum creatine phosphokinase level was normal. X‐rays demonstrated lumbar spondylosis but no scoliosis or kyphosis. Electrocardiography and echocardiography revealed normal patterns. Pulmonary function tests demonstrated moderate obstructive and mild restrictive ventilatory defects, with an forced vital capacity of 3.8 L (66% of reference) as well as decreased diffusion capacity.

## PATHOLOGIC FINDINGS

**Figure 3:**
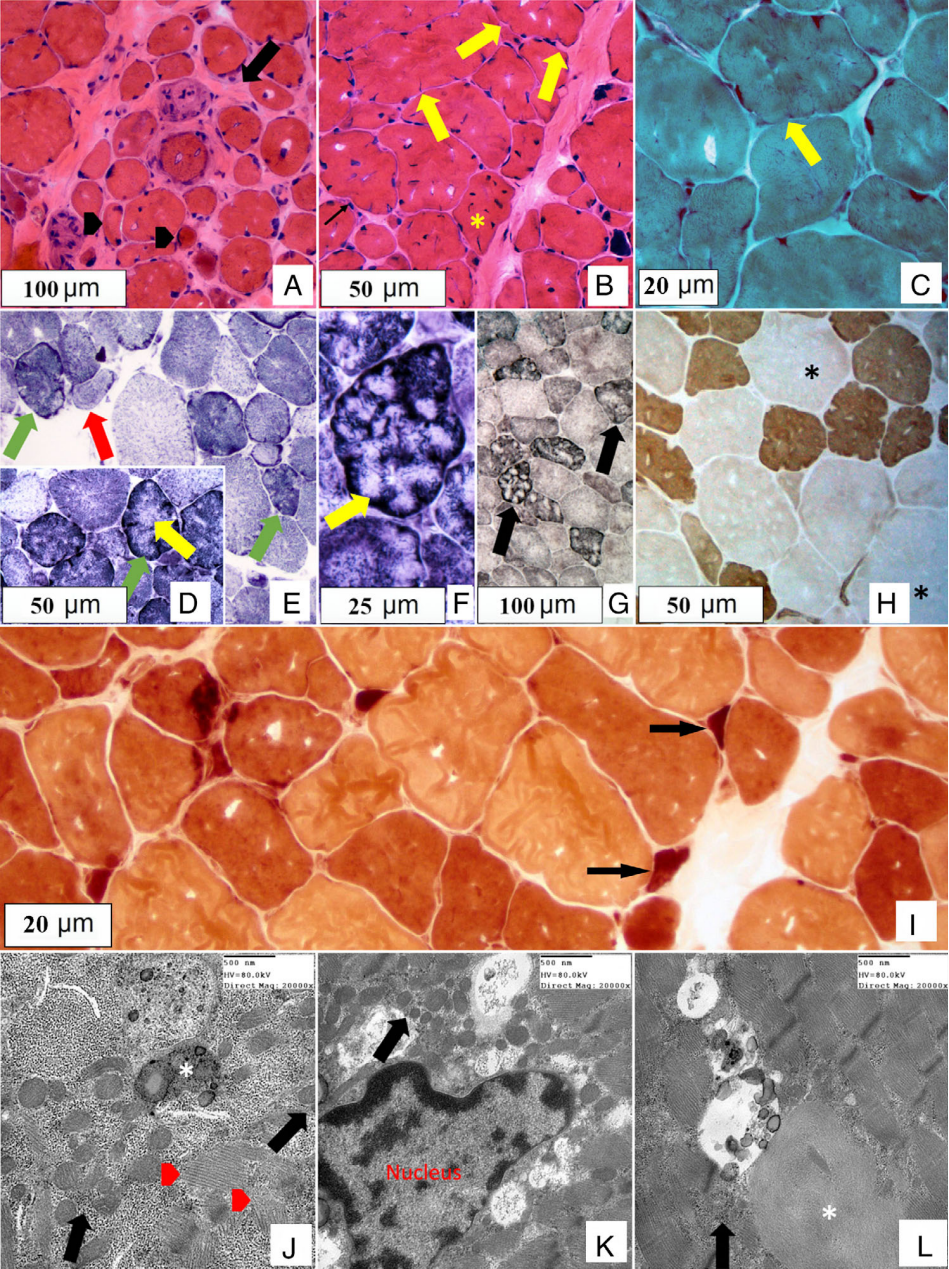
Patient II‐1's muscle biopsy of the biceps brachii at 37 years. (A) Hematoxylin and eosin (HE) staining demonstrates fibrosis (arrow) and fiber atrophy (arrowheads) causing fascicular disarray. (B) HE demonstrates internal nucleation (asterisk), fiber atrophy/hypertrophy, and sarcolemmal notching (arrows). (C) Modified Gomori‐trichrome staining demonstrates trabecular changes with sarcolemmal notching (yellow arrow). (D) Nicotinamide adenine dinucleotide tetrazolium reductase (NADH‐TR) reaction indicates a fiber with zones of dense aggregation alternating with areas of lucency (green arrow) and core features (yellow arrow). (E) By NADH‐TR reaction, subsarcolemmal aggregates and sarcoplasmic irregularity; both ring fibers with circumferential striation (red arrows) and end‐stage atrophic fibers with subsarcolemmal aggregation of the organelles (green arrows) are also evident. (F) NADH‐TR reaction demonstrates core features (F, yellow arrow). (G) Succinate dehydrogenase (SDH) reaction demonstrates subsarcolemmal aggregates consistent with mitochondria. Both NADH‐TR and SDH reactions demonstrate fiber lobulation (trabecular morphology). (H) Myosin adenosine triphosphatase pH 4.2 shows type 1 fiber atrophy (dark fibers) and sarcolemmal notching, and type 2 fiber hypertrophy (asterisk); the mosaic pattern of fiber type distribution is preserved. (I) Non‐specific esterase reaction shows scattered angular atrophy (arrows). (J) Electron microscopy reveals subsarcolemmal mitochondrial aggregation (arrow), myofibrillar disorganization (arrowhead) and lipofuscin (asterisk). (K) Electron microscopy reveals perinuclear areas of mitochondrial aggregation and hyperplasia (arrow). (L) Electron microscopy reveals myofibrillar separation and disarray in central areas of the myofiber and interruption by lipid and lipofuscin (arrow); note the presence of a filamentous body (approximately 2 × 3 μm in size) (asterisk). No autophagic vacuoles or excess storage material is identified. There are no mitochondrial structural abnormalities. Cyclooxygenase (COX) staining demonstrates type‐1 fibers with a “notched” contour and a trabecular morphology (asterisk). While COX staining is adequate for diagnostic purposes the quality of the slide degraded with time and is unsuitable for publication. An image for the COX staining is available in Figure S1.

Biopsy of left biceps brachii at 37 years of age showed displacement of the fascicular architecture with patchy endomysial compartment fibrosis (Fig. 3). Internal nucleation was prominent (often 3–8 nuclei per fiber). There was no significant inflammation, degenerating/regenerating myofibers or split fibers. Fiber size variation was noted with round atrophy and scattered hypertrophic fibers. There were no rimmed vacuoles. Trabecular changes, sarcolemmal notching, cytoplasmic cores, and subsarcolemmal aggregates were prominent throughout the biopsy. Occasional ring fibers were also noted. These features are demonstrated in Figure 3D‐F. There was no evidence of significant denervation on non‐specific esterase to account for the extent of these cytoplasmic changes, as these features may be seen in association with advanced chronic neurogenic syndromes. The stain revealed occasional hyperstained angular atrophic fibers associated with very rare sarcolemmal nuclear aggregates. Myosin adenosine triphosphatase with appropriate pH differentiation confirmed prominent fiber size variation with preferential type‐1 fiber smallest, scattered type‐2 fiber hypertrophy and preservation of the mosaic architecture. The intermediate sized type‐1 fibers had a “notched” contour and a trabecular morphology on oxidative and dehydrogenase reactions (nicotinamide adenine dinucleotide, succinate dehydrogenase, cyclooxygenase (COX).^5^, ^6^ (see COX staining in Fig. S1). Muscle immunohistochemical staining for dystrophin, dysferlin, merosin, alpha‐, beta‐ and gamma‐sarcoglycans, and desmin were all normal.

In correlation with histochemical findings, electron microscopy at multiple direct magnifications revealed subsarcolemmal and perinuclear areas of mitochondrial hyperplasia and aggregation (Fig. 3). There were no mitochondrial structural abnormalities. Myofibrillar disorganization was noted throughout the myofiber. This consisted mainly of sarcomeric discontinuation and small haphazardly distributed bundles of sarcomeres. Rare filamentous bodies were also observed. No autophagic vacuoles or excess storage material was identified.

The proband was given a diagnosis of trabecular myopathy of an unspecified genetic etiology at the age of 37 years. Facioscapulohumeral muscular dystrophy genetic testing was normal. At the age of 43 years, clinical exome sequencing was performed, and no pathogenic variants were detected for any known myopathy. As such, the patient was enrolled in the Undiagnosed Diseases Network (UDN) clinical site at the University of California Los Angeles (UCLA).

## GENETIC ANALYSIS

Patient II‐1 consented and was enrolled in the UCLA UDN after he had non‐diagnostic re‐evaluation of his prior trio exome sequencing. Whole genome sequencing was performed on the proband, both parents and his similarly affected maternal aunt (I‐3) at the Hudson‐Alpha UDN sequencing core. All sequenced family members had high‐quality genomic sequences falling within normal human genomic variation quality parameters.

Sequencing in the proband detected several regions of homozygosity spanning approximately 13% of the genome, as expected given family history of multiple consanguinity. Genome sequencing identified homozygous pathogenic variants in *PYROXD1* (NM_024854.5:c.464A>G [p.Asn155Ser]) in both the proband and I‐3, and directed sequencing observed homozygosity for individual I‐4 as well. This variant was classified as pathogenic based on the prior report of O'Grady *et al*.^1^

## DISCUSSION

Myopathy associated with *PYROXD1* pathogenic variants has now been reported in a total of 17 individuals, including the three reported here. The phenotype of this disorder is generally consistent and causes a slowly progressive myopathy. However, there is phenotypic variation that must be taken into account when counseling affected individuals. O'Grady *et al*. originally reported facial weakness, nasal speech and hyporeflexivity as universal features in the first nine patients with *PYROXD1*‐related autosomal recessive myopathy.^1^ However, these features have not been consistently reported in other patients with *PYROXD1*‐associated myopathy.^2^, ^3^, ^4^

Patient II‐1 was under observation in a neuromuscular clinic for over 10 years prior to genetic diagnosis and is best characterized by slowly progressive proximal myopathy with periodic myalgia. Similar to the previously reported patients, he had normal cognitive function. Patients I‐3 and I‐4 both had evidence of slowly progressive proximal myopathy with onset in the fifth decade of life. Both are alive and ambulatory, without use of respiratory support at 78 and 82 years of age which indicates that *PYROXD1*, at least with the now common causal allele, p.Asn155Ser does not necessarily dramatically shorten lifespan.

Imaging, echocardiography and pulmonary function testing of Patient II‐1 confirmed similarities between the 10 reported patients with *PYROXD1*‐related myopathy. Patient II‐1 had relative sparing of the rectus femoris and hip adductor group from atrophy on MRI consistent with MRI findings of other affected patients.^1^, ^3^ Therefore, sparing of these muscle groups may be a common sign in *PYROXD1*‐associated myopathy.

O'Grady *et al*. described one patient with possible cardiomyopathy and Lornage *et al*. described one patient with mild septal and anteroseptal dyskinesia.^1^, ^4^ However, the majority of patients with *PYROXD1*‐associated myopathy (including the patients reported in this paper) did not show signs of cardiomyopathy even at advanced age.^1^, ^2^, ^3^ Thus, while future patients may benefit from periodic monitoring for cardiomyopathy, cardiomyopathy does not appear to be common in *PYROXD1*‐associated myopathy. Pulmonary function tests in Patient II‐1 demonstrated a mild obstructive and possible restrictive pattern as well as decreased diffusion capacity. This finding is consistent with other reports of a restrictive pattern of pulmonary function in other cases of *PYROXD1*‐associated myopathy.^1^, ^2^, ^3^, ^4^ Lornage *et al*. also reported two patients who were ventilator dependent by ages 14 and 15, which demonstrates the range of ventilatory impairment that can be seen in *PYROXD1*‐associated myopathy.^4^

Lornage *et al*. also reported that patients with splice‐site altering variants have decreased *PYROXD1* transcription and/or PYROXD1 protein along with a severe and early‐onset form of the disease.^4^ Therefore, there may be a genotype–phenotype correlation with splice‐site variants in *PYROXD1* leading to more severe disease and missense variants such as p.Asn155Ser leading to less severe disease. However, given the small number of patients reported with *PYROXD1*‐associated myopathy caution should be utilized when drawing conclusions about genotype–phenotype correlations in this rare disease.

Muscle biopsy revealed a complex pathology including prominent trabecular changes, sarcoplasmic rearrangement of mitochondria without structural abnormality and subtle myofibrillar disorganization. The presence of trabecular fibers in this biopsy and subsequent finding of *PYROXD1* mutation is noteworthy. These fibers may represent diagnostic features of *PYROXD1*‐associated myopathy and expand the realm of differentials associated with such a histologic phenotype. The possibility of *PYROXD1* mutation as one of the causes of historically defined “trabecular fiber myopathy” is herein proposed but requires additional studies of the relevant patient population.

## FUNDING

Research reported in this manuscript was supported by the National Institute of Health (NIH) Common Fund, through the Office of Strategic Coordination/Office of the NIH Director under Award Numbers U01HG007703, U01HG007672, U01HG007690, U01HG007674, and U01HG007530. Jeremy Woods was supported by a USHHS Ruth L. Kirschstein Institutional National Research Service Award (T32GM008243).

## DISCLOSURE

The authors declare no conflict of interest.

## Supporting information


**Figure S1** Cyclo‐oxygenase (COX) staining demonstrated type‐1 fibers with a “notched” contour and a trabecular morphology.
**Table S1** Table of phenotypic and genotypic data of patients previously reported with *PYROXD1*‐associated myopathy.
**Appendix S1** Supplementary Information.
