# Supplementary material for: Myopathy associated with homozygous PYROXD1 pathogenic variants detected by genome sequencing
Source: Neuropathology. 2020 Feb 9;40(3):302–7. doi: 10.1111/neup.12641 (PMC7317439; doi:10.1111/neup.12641)
Supplement: Supplementary file 1 — Figure S1 Cyclo‐oxygenase (COX) staining demonstrated type‐1 fibers with a “notched” contour and a trabecular morphology. Table S1 Table of phenotypic and genotypic data of patients previously reported with PYROXD1‐associated myopathy. Appendix S1 Supplementary Information. [file NEUP-40-302-s001.zip › NEUP_12641_Patient Table Redux.pdf]

	Sex	Ancestry	PYROXD1 Variant	Age at onset (years)	Symptoms at Onset
**Saha et al.**	F	Arab	Homozygous c.464A>G, p.Asn155Ser	9	Progressive weakness with falling.
**Sainio et al.**	M	Finnish	Homozygous c.464A>G, p.Asn155Ser	10	Difficulty going up stairs, running.
**Sainio et al.**	M	Finnish	Homozygous c.464A>G, p.Asn155Ser	30	Difficulty rising from sitting.
**Sainio et al.**	F	Finnish	Homozygous c.464A>G, p.Asn155Ser	33	Difficulty rising from sitting.
**Sainio et al.**	M	Finnish	Compound heterozygous c.464A>G, p.Asn155Ser & c.1061A>G, p.Tyr354Cys	49	Slowly progressive proximal muscle weakness.
**O'Grady et al.**	M	European	Compound heterozygous c.285+1G>G & c.1116G>C, p.Glu372His	5	Difficulty running.
**O'Grady et al.**	M	European	Compound heterozygous c.285+1G>G & c.1116G>C, p.Glu372His	8	Difficulty with stairs.
**O'Grady et al.**	M	Turkish	Homozygous c.464A>G, p.Asn155Ser	10	Difficulty ascending stairs.
**O'Grady et al.**	M	Turkish	Homozygous c.464A>G, p.Asn155Ser	10	n/a
**O'Grady et al.**	F	Persian Jewish	Compound heterozygous c.414+1G>A & c.464A>G, p.Asn155Ser	Congenital	Hypotonia, walked at 20 months.

**O'Grady et al.**	M	Persian Jewish	Compound heterozygous c.414+1G>A & c.464A>G, p.Asn155Ser	Infancy	Hypotonia, walked at 13 months.
**O'Grady et al.**	F	Turkish	Homozygous c.464A>G, p.Asn155Ser	2	Difficulty running and climbing with frequent falls.
**O'Grady et al.**	M	Turkish	Homozygous c.464A>G, p.Asn155Ser	2.5	Difficulty climbing.
**O'Grady et al.**	F	Turkish	Compound heterozygous c.1159_1160insCAAA, p.Ala387fs & c.464A>G, p.Asn1555Ser	4	Fatigue, falls, difficulty with stairs.
**Lornage et al.**	M	Not reported	Compound heterozygous c.285 + 1G > A & c.464A>G p.Asn155Ser	Congenital	Axial weakness.
**Lornage et al.**	M	Not reported	Homozygous c.464A>G, p.Asn155Ser	Childhood	Axial, upper and lower limb weakness.
**Lornage et al.**	F	Not reported	Compound heterozygous c.415-976A>G & c.1116G>C, p.Gln372His	Congenital	Diffuse weakness.
**Woods et al.**	M	Persian Jewish	Homozygous c.464A>G, p.Asn155Ser	30-33	Difficulty ascending stairs, throwing balls. Muscle pain.
**Woods et al.**	F	Persian Jewish	Homozygous c.464A>G, p.Asn155Ser	50s	Difficulty walking, ascending stairs, rising from chairs. Muscle cramps.
**Woods et al.**	M	Persian Jewish	Homozygous c.464A>G, p.Asn155Ser	50s	Difficulty walking, ascending stairs, rising from chairs. Muscle cramps.

	**Ambulatory Status**	**Histopathology**	**CK (U/L)**	**Imaging Studies**
**Saha et al.**	Wheelchair bound at 37.	No biopsy	74	None

**Sainio et al.**	Ambulant at 64 with walking sticks.	Dystrophic	288-340	Bilateral fibrofatty replacement of all extremity muscles with the dorsal muscle most severely affected.
**Sainio et al.**	Ambulant at 70 with walking sticks.	Dystrophic	normal	Diffuse fatty replacement in gluteal, thigh and leg muscles.
**Sainio et al.**	Wheelchair bound at 66.	Dystrophic	normal	Diffuse fatty replacement of gluteal, thigh and leg muscles at 65 years.
**Sainio et al.**	Still ambulant at 63.	Dystrophic	normal	Bilateral atrophy and fatty replacement in lower limb muscle, atrophy in upper limbs greater than atrophy in pectoral girdle muscles.
**O'Grady et al.**	Slow walk with cane at 24	Fiber size variation with fibrosis. Internalized nuclei with sarcomeric disorganization.	148-262	None
**O'Grady et al.**	unknown	No biopsy	118-1,051	MRI thigh and calf with diffuse muscle atrophy and fatty marbling with relative sparing of the rectus femoris.
**O'Grady et al.**	unknown	No biopsy	500-700	MRI thigh and calf with diffuse muscle atrophy and fatty marbling with relative sparing of the rectus femoris. Thigh less affected than calf.
**O'Grady et al.**	unknown	Fiber size variation with fibrosis. Internalized nuclei with sarcomeric disorganization.	700-800	MRI thigh and calf with diffuse muscle atrophy and fatty marbling with relative sparing of the rectus femoris. Thigh less affected than calf.
**O'Grady et al.**	unknown	Fiber size variation with fibrosis. Internalized nuclei with sarcomeric disorganization.	normal	None
**O'Grady et al.**	unknown	No biopsy	normal	MRI thigh and calf with diffuse muscle atrophy and fatty marbling with relative sparing of the rectus femoris.
**O'Grady et al.**	unknown	Fiber size variation with fibrosis. Internalized nuclei with sarcomeric disorganization.	400-700	None
**O'Grady et al.**	unknown	No biopsy	290-376	None
**O'Grady et al.**	unknown	Fiber size variation with fibrosis. Internalized nuclei.	normal	None
**Lornage et al.**	Wheelchair bound at 12.	Fiber size variation with fibrosis, internalized nuclei and cores.	Not reported	Whole body MRI with diffuse fatty infiltration, most prominent in gluteus maximus and vastus lateralis/intermedius/medius.
**Lornage et al.**	unknown	Internalized nuclei, cores and myofibrillar inclusions.	Not reported	
**Lornage et al.**	Wheelchair bound at 12.	Internalized nuclei, cores, rods and myofibrillar inclusions.	Not reported	MRI with diffuse fatty infiltration, most prominent in gluteus maximus and vastus lateralis/intermedius/medius.

**Woods et al.**	Still ambulant at 47.	Fibrosis with fiber atrophy and fascicular disarray.	158	MRI thigh with diffuse muscle atrophy and fatty marbling with relative sparing of the rectus femoris and thigh adductor group.
**Woods et al.**	Still ambulant at 82.	No biopsy	n/a	None
**Woods et al.**	Still ambulant at 78.	No biopsy	n/a	None

	**Respiratory**	**Cardiac**	**Swallowing/Speech**	**Deep Tendon Reflexes**	**Misc.**
**Saha et al.**	Not reported.	No problems reported.	No problems reported.	Normal	Two C-sections for failure to progress.
**Sainio et al.**	FVC 40% predicted (64 years).	Echocardiography normal at 60 years.	No problems reported.	Not reported	Brother also had unknown muscle disease and died of pneumonia at 43.
**Sainio et al.**	FVC 67% with reduced MIP, MEP and PCF (70 years).	Echocardiography normal at 66 years.	Hoarse voice.	Not reported	Weak cough.
**Sainio et al.**	FVC 42% and 30% predicted at 59 and 68 years.	No problems reported.	No problems reported.	Not reported	Death at 70 secondary to pneumonia and respiratory insuficiency.
**Sainio et al.**	FVC 54% predicted (63 years).	Normal	No problems reported.	Not reported	Diagnosed with pulmonary sarcoidosis.
**O'Grady et al.**	Restrictive lung disease by 15 years.	Low ejection fraction with septal wall motion abnormality by 27.	Dysphagia, facial weakness and nasal speech.	Decreased or absent.	Diffusely decreased muscle bulk with scapular winging. Feet with pes cavus and skin discoloration. High arched palate. Thoracic scoliosis.
**O'Grady et al.**	No	No	Dysphagia, facial weakness and nasal speech.	Decreased or absent.	Hypothenar and thenar muscle wasting. High arched palate.
**O'Grady et al.**	No	No	Dysphagia, facial weakness and nasal speech.	Decreased or absent.	

**O'Grady et al.**	Yes	No	Dysphagia and facial weakness.	Decreased or absent.	
**O'Grady et al.**	No	Pulmonic valve insufficiency.	Dysphagia and facial weakness.	Decreased or absent.	Diffuse muscle wasting and hyperextensibility at wrist and elbow. High arched palate. Scoliosis.
**O'Grady et al.**	No	No	Dysphagia and facial weakness.	Decreased or absent.	Hand and wrist hyperextension. High arched palate.
**O'Grady et al.**	No	No	Dysphagia, facial weakness and nasal speech.	Decreased or absent.	High arched palate.
**O'Grady et al.**	No	No	Dysphagia, facial weakness and nasal speech.	Decreased or absent.	Stable in childhood. High arched palate.
**O'Grady et al.**	No	Mitral & tricuspid valve regurgitation.	Dysphagia, facial weakness and nasal speech.	Decreased or absent.	High arched palate.
**Lornage et al.**	Non-invasive ventilation with oxygen starting at 14 years.	No problems reported.	No nasal speech.	Not reported	Joint hypermobility & scoliosis.
**Lornage et al.**	Vital capacity 68% predicted (age unknown)	No problems reported.	No problems reported.	Not reported	No scoliosis
**Lornage et al.**	Non-invasive ventilation with oxygen starting at 15 years.	Mild septal and anteroseptal dyskinesia.	Nasal speech.	Not reported	Scoliosis, high-arched feet, hand asymmetry, low-set ears, decreased bone mineral density.
**Woods et al.**	FVC of 3.8L (66% of reference) and decreased diffusion capacity.	Normal EKG and echocardiography	No problems reported.	Normal	
**Woods et al.**	None reported.	No problems reported.	No problems reported.	Not reported	
**Woods et al.**	None reported.	No problems reported.	No problems reported.	Not reported	
