# Supplementary material for: Myopathy associated with homozygous PYROXD1 pathogenic variants detected by genome sequencing
Source: Neuropathology. 2020 Feb 9;40(3):302–7. doi: 10.1111/neup.12641 (PMC7317439; doi:10.1111/neup.12641)
Supplement: Supplementary file 1 — Figure S1 Cyclo‐oxygenase (COX) staining demonstrated type‐1 fibers with a “notched” contour and a trabecular morphology. Table S1 Table of phenotypic and genotypic data of patients previously reported with PYROXD1‐associated myopathy. Appendix S1 Supplementary Information. [file NEUP-40-302-s001.zip › NEUP_12641_UDN Corporate Authors.pdf]

Last	First
Acosta	Maria
Adams	David
Aday	Aaron
Alejandro	Mercedes
Allard	Patrick
Ashley	Euan
Azamian	Mahshid
Bacino	Carlos
Baker	Eva
Balasubramanyam	Ashok
Batzli	Gabriel
Beggs	Alan
Bellen	Hugo
Bernstein	Jonathan
Berry	Gerard
Bican	Anna
Bick	David
Birch	Camille
Bonnenmann	Carsten
Bonner	Devon
Boone	Braden
Bostwick	Bret
Briere	Lauren
Brokamp	Elly
Brown	Donna
Brush	Matthew
Burke	Elizabeth
Burrage	Lindsay
Butte	Manish
Chao	Hsiao-Tuan
Clark	Gary
Coakley	Terra
Cogan	Joy
Colley	Heather
Cooper	Cynthia
Cope	Heidi

Craigen	William
D'Souza	Precilla
Davids	Mariska
Davidson	Jean
Dayal	Jyoti
Dell'Angelica	Esteban
Dhar	Shweta
Donnell-Fink	Laurel
Dorrani	Naghmeh
Dorset	Daniel
Douine	Emilie
Draper	David
Dries	Annika
Duncan	Laura
Eckstein	David
Emrick	Lisa
Eng	Christine
Enns	Gregory
Esteves	Cecilia
Estwick	Tyra
Fernandez	Liliana
Ferreira	Carlos
Fieg	Elizabeth
Fisher	Paul
Fogel	Brent
Friedman	Noah
Gahl	William
Godfrey	Rena
Goldman	Alica
Goldstein	David
Gourdine	Jean-Philippe
Groden	Catherine
Gropman	Andrea
Haendel	Melissa
Hamid	Rizwan
Hanchard	Neil
High	Frances

Holm	Ingrid
Hom	Jason
Huang	Yong
Huang	Alden
Jamal	Fariha
Jiang	Yong-hui
Johnston	Jean
Jones	Angela
Karaviti	Lefkothea
Kelley	Emily
Koeller	David
Kohane	Isaac
Kohler	Jennefer
Korrick	Susan
Kozuira	Mary
Krakow	Deborah
Krasnewich	Donna
Krier	Joel
Kyle	Jennifer
Lalani	Seema
Lau	C.
Lazar	Jozef
LeBlanc	Kimberly
Lee	Brendan
Lee	Hane
Levy	Shawn
Lewis	Richard
Lincoln	Sharyn
Liu	Pengfei
Loo	Sandra
Loscalzo	Joseph
Maas	Richard
Macnamara	Ellen
MacRae	Calum
Maduro	Valerie
Majcherska	Marta
Malicdan	May Christine

Mamounas	Laura
Manolio	Teri
Markello	Thomas
Marom	Ronit
Martin	Martin
Martínez-Agosto	Julian
Marwaha	Shruti
May	Thomas
McConkie-Rosell	Allyn
McCormack	Colleen
McCray	Alexa
Merker	Jason
Metz	Thomas
Might	Matthew
Moretti	Paolo
Morimoto	Marie
Mulvihill	John
Murdock	David
Nath	Avi
Nelson	Stanley
Newberry	J.
Newman	John
Nicholas	Sarah
Novacic	Donna
Orengo	James
Pallais	J.
Palmer	Christina
Papp	Jeanette
Parker	Neil
Pena	Loren
Phillips III	John
Posey	Jennifer
Postlethwait	John
Potocki	Lorraine
Pusey	Barbara
Renteria	Genecee
Reuter	Chloe

Rives	Lynette
Robertson	Amy
Rodan	Lance
Rosenfeld	Jill
Rowley	Robb
Sampson	Jacinda
Samson	Susan
Schoch	Kelly
Scott	Daryl
Shakachite	Lisa
Sharma	Prashant
Shashi	Vandana
Signer	Rebecca
Sillari	Catherine
Silverman	Edwin
Sinsheimer	Janet
Smith	Kevin
Spillmann	Rebecca
Stoler	Joan
Stong	Nicholas
Sullivan	Jennifer
Sweetser	David
Tamburro	Cecelia
Tan	Queenie
Tifft	Cynthia
Toro	Camilo
Tran	Alyssa
Urv	Tiina
Vogel	Tiphanie
Waggott	Daryl
Wahl	Colleen
Walker	Melissa
Walley	Nicole
Walsh	Chris
Wan	Jijun
Wang	Lee-kai
Wangler	Michael
Ward	Patricia

Waters	Katrina
Webb-Robertson	Bobbie-Jo
Westerfield	Monte
Wheeler	Matthew
Wise	Anastasia
Wolfe	Lynne
Woods	Jeremy
Worthey	Elizabeth
Yamamoto	Shinya
Yang	John
Yoon	Amanda
Yu	Guoyun
Zastrow	Diane
Zhao	Chunli
