# Supplementary figures and images for: Myopathy associated with homozygous PYROXD1 pathogenic variants detected by genome sequencing
Source: Neuropathology. 2020 Feb 9;40(3):302–7. doi: 10.1111/neup.12641 (PMC7317439; doi:10.1111/neup.12641)

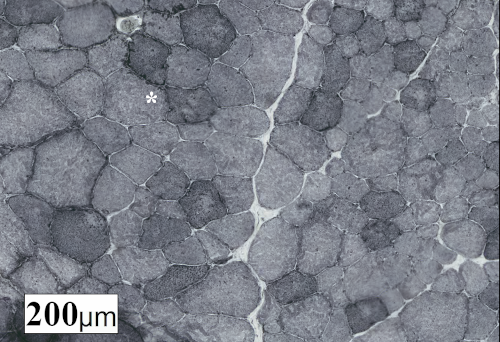

Supplement: Supplementary file 1 — Figure S1 Cyclo‐oxygenase (COX) staining demonstrated type‐1 fibers with a “notched” contour and a trabecular morphology. Table S1 Table of phenotypic and genotypic data of patients previously reported with PYROXD1‐associated myopathy. Appendix S1 Supplementary Information. [file NEUP-40-302-s001.zip › NEUP_12641_PYROXD1 COX.tif]
